# Treatment Outcomes of Computer Tomography-Guided Brachytherapy in Cervical Cancer in Hong Kong: A Retrospective Review

**DOI:** 10.3390/cancers14163934

**Published:** 2022-08-15

**Authors:** Wing-Lok Chan, Matthew Ho-Fai Cheng, Jacky Tsun-Kit Wu, Cheuk-Wai Choi, Rosa Piu-Ying Tse, Patty Piu-Ying Ho, Emina Edith Cheung, Andy Cheung, Ka-Yu Test, Karen Kar-Loen Chan, Hexane Yuen-Sheung Ngan, Steven Wai-Kwan Siu, Roger Kai-Cheong Ngan, Anne Wing-Mui Lee

**Affiliations:** 1 Department of Clinical Oncology, LKS Faculty of Medicine, The University of Hong Kong, Hong Kong, China; 2 LKS Faculty of Medicine, The University of Hong Kong, Hong Kong, China; 3 School of Public Health, LKS Faculty of Medicine, The University of Hong Kong, Hong Kong, China; 4 Department of Clinical Oncology, Queen Mary Hospital, Hong Kong, China; 5 Department of Obstetrics and Gynecology, LKS Faculty of Medicine, The University of Hong Kong, Hong Kong, China; 6 Department of Clinical Oncology, Gleneagles Hospital, Hong Kong, China; 7 Department of Clinical Oncology, The University of Hong Kong-Shenzhen Hospital, Shenzhen 518009, China

**Keywords:** cervical cancer, image-guided brachytherapy, long-term outcome, adenocarcinoma, local control, computer tomography, survival

## Abstract

**Simple Summary:**

This retrospective study reviews 135 patients with locally advanced cervical cancer treated with chemo-radiotherapy with image-guided adaptive brachytherapy with CT guidance. The study has a long follow-up period of 53.6 months. The outcome was excellent with a five-year local control, pelvic control, distant metastasis-free survival and overall survival rates being 90.7%, 84.2%, 80.0% and 87.2%, respectively. Adenocarcinoma was significantly associated with worse local control, pelvic control, distant metastasis-free survival and overall survival rates.

**Abstract:**

(1) Background: To report the long-term clinical outcomes of computer-tomography (CT)-guided brachytherapy (BT) for locally advanced cervical cancer. (2) Methods: A total of 135 patients with FIGO stage IB-IVA cervical cancer treated with definitive radiotherapy +/− chemotherapy with an IGABT boost at Queen Mary Hospital, Hong Kong, between November 2013 and December 2019 were included. Treatment included pelvic radiotherapy 40 Gy/20 Fr/4 weeks +/− chemotherapy then CT-guided BT (7 Gy × 4 Fr) and a sequential parametrial boost. The primary outcome was local control. Secondary outcomes were pelvic control, distant metastasis-free survival, overall survival (OS) and late toxicities. (3) Results: The median follow-up was 53.6 months (3.0–99.6 months). The five-year local control, pelvic control, distant metastasis-free survival and OS rates were 90.7%, 84.3%, 80.0% and 87.2%, respectively. The incidence of G3/4 long-term toxicities was 6.7%, including proctitis (2.2%), radiation cystitis (1.5%), bowel perforation (0.7%), ureteric stricture (0.7%) and vaginal stenosis and fistula (0.7%). Patients with adenocarcinomas had worse local control (HR 5.82, 95% CI 1.84–18.34, *p* = 0.003), pelvic control (HR 4.41, 95% CI 1.83–10.60, *p* = 0.001), distant metastasis-free survival (HR 2.83, 95% CI 1.17–6.84, *p* = 0.021) and OS (HR 4.38, 95% CI: 1.52–12.67, *p* = 0.003) rates. Distant metastasis-free survival was associated with HR-CTV volume ≥ 30 cm^3^ (HR 3.44, 95% CI 1.18–9.42, *p* = 0.025) and the presence of pelvic lymph node (HR 3.44, 95% CI 1.18–9.42, *p* = 0.025). OS was better in patients with concurrent chemotherapy (HR 4.33, 95% CI: 1.40–13.33, *p* = 0.011). (4) Conclusions: CT-guided BT for cervical cancer achieved excellent long-term local control and OS. Adenocarcinoma was associated with worse clinical outcomes. (4) Conclusion: CT-guided BT for cervical cancer achieved excellent long-term local control and OS. Adenocarcinoma was associated with worse clinical outcomes.

## 1. Introduction

Cervical cancer is the fourth most commonly diagnosed cancer and the fourth leading cause of cancer death in women, with an estimated 604,000 new cases and 342,000 deaths worldwide in 2020 [1]. The incidence rate has declined in the past two decades in some countries because of the highly effective primary (HPV vaccine) and secondary (screening) prevention measures. However, in some parts of the world, e.g., Eastern Europe and Central Asia, the incidence of cervical cancer is still rising. It is estimated the incidence rate and mortality rate will increase by 40.2% and 53.4%, respectively, in 2040, making it a major public health problem [2]. In Hong Kong, cervical cancer ranked eighth in terms of both cancer incidence and cancer mortality in 2019 [3]. Most patients present with locally advanced tumors, for which curative surgery is not possible.

The standard treatment for locally advanced cervical cancer is concurrent chemo-radiotherapy (CRT), followed by a boost to the primary tumor with brachytherapy (BT). BT plays a crucial role in the management of cervical cancer and is associated with pelvic control and overall survival (OS). Image-guided adaptive brachytherapy (IGABT) with magnetic resonance imaging (MRI) or computer tomography (CT) guidance, using three-dimensional (3D) instead of two-dimensional (2D) planning, can increase the target-coverage conformity, allow dose escalation and decrease doses to the organ-at-risks (OARs). IGABT has been recommended in different international associations, including the Gynaecological (GYN) GEC-ESTRO Working Group, American Brachytherapy Society, and the Royal College of Radiologists [4,5,6,7]. EMBRACE-I, a prospective, observational, multicenter cohort study, which included 1416 patients confirmed that MRI-based IGABT, resulted in long-term local control across all stages of locally advanced cervical cancer (overall five-year local control: 92%) [8]. Moreover, several retrospective studies from mono-institution studies and the multi-institutional RetroEMBRACE study reported the excellent local control of 85–95%, with limited toxicities [9,10,11,12].

MRI is the gold standard of imaging for IGABT because of its superior soft-tissue contrast and more accurate target delineation relative to CT. However, its wide applicability is limited by its availability, logistics and financial implications. CT is an attractive alternative to MRI because of its wide availability in most of the radiation departments and much lower cost. Various surveys also reported that CT utilization was higher than MRI for planning BT in cervical cancers [13,14,15,16].

CT-based IGABT for gynecological cancers was introduced at Queen Mary Hospital, Hong Kong in November 2013. The aim of this study is to analyze the data in our institution to determine the long-term clinical outcomes of CT-based IGABT for locally advanced cervical cancer.

## 2. Materials and Methods

### 2.1. Study Design

All patients with histologically confirmed International Federation of Gynaecology and Obstetrics (FIGO) stage IB-IVA cervical cancer (2009 staging system) treated with definitive radiotherapy +/− chemotherapy with an IGABT boost at Queen Mary Hospital, Hong Kong, between November 2013 and December 2019 were included [17].

All patients underwent clinical staging according to FIGO criteria by both a gynecologist and clinical oncologist. After a physical examination, all patients underwent pelvic MRI for local staging, and CT thorax and abdomen or positron emission tomography (PET)–CT for any distant metastasis. The clinical tumor size was defined as the maximum width of the palpable mass on pelvic examination. Tumor size on the MRI was defined as the maximum width on axial T2-weighted sequences. Tumor depth was the depth of invasion on coronal views. Pathological lymph nodes were defined as lymph nodes over 1 cm in size or a positive uptake on PET-CT.

### 2.2. Treatment Delivery

#### 2.2.1. Pelvic Radiotherapy

**Figure 1:**
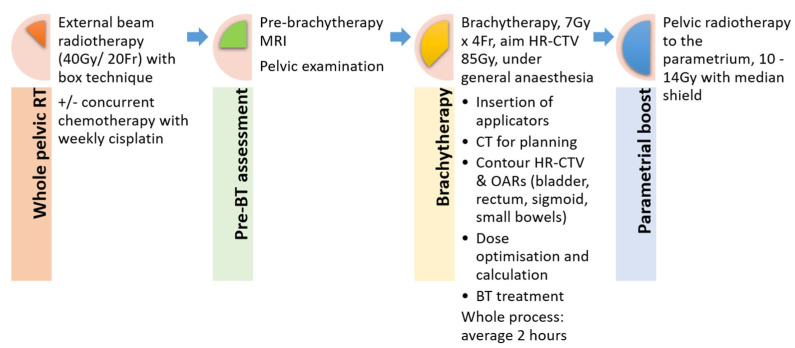
Workflow of chemo-radiotherapy for cervical cancer (whole pelvic radiotherapy then reassessment with MRI, followed by brachytherapy and external beam radiotherapy for parametrial boost).

All patients received radiotherapy (RT) to the whole pelvis with a 3D conformal box technique at a dose of 40 Gy in 20 fractions over 4 weeks with or without concurrent chemotherapy and weekly administration of cisplatin at 40 mg/m^2^. Four fractions of BT was given afterwards. Then, a sequential RT boost to parametrium, generally 10 to 14 Gy (at 2 Gy per fraction) with midline shielding, was used individually where cumulative dose coverage achieved from EBRT and BT was not adequate (Figure 1).

#### 2.2.2. Brachytherapy

All patients had pelvic examinations and pre-BT MRIs for response assessments during the last week of chemoradiation. Brachytherapy was conducted immediately after pelvic RT in 4 fractions over 2 weeks (Monday and Thursday). The aim was to deliver 7 Gy for a high-risk clinical target volume (HR-CTV) per fraction (7 Gy × 4). CT/MR compatible Elekta Utrecht applicators were used in November 2013 to October 2015. The possibility of interstitial needle insertion was implemented from October 2015. After December 2017, the more modern Venezia applicator with the possibility of inserting oblique needles was adopted.

Planning CT was performed following applicator insertion. The bladder was filled with 100 mL of water at the time of CT and before treatment to ensure the same bladder volume. The HR-CTV and OARs (bladder, rectum, sigmoid and small bowels) were contoured for each insertion according to the GEC-ESTRO guidelines adapted to CT brachytherapy planning and the NRG Oncology Atlas [18]. The HR-CTV included all the tumors with reference from the clinical examination and MRI findings before brachytherapy. It was contoured at the level of the applicator to the uterus indents, laterally covering the parametrial extension. Brachytherapy treatment planning was conducted on Oncentra Brachytherapy versions 4.4–4.6 over the study period. Dose optimization and planning were completed after each insertion.

For each brachytherapy dosimetry plan, the following dose parameters were recorded: HR-CTV D90 (the isodose encompassing 90% of the tumor target); V100 of HR-CTV (the percentage of tumor target volume receiving 100% of the prescribed dose); and D2cc (minimum dose in the most exposed 2 cm^3^ volume) for bladder, rectum, sigmoid and bowel. The EBRT and BT doses were summated to a biologically equivalent dose in a 2 Gy/fraction (EQD_2_) using the linear quadratic model with α/β equal to 10 Gy for tumor effects, α/β equal to 3 Gy for late normal tissue damage and a repair half-time of 1.5 h for a high-dose rate. The dose aimed to achieve HR-CTV D90 EQD_2_ ≥ 85 Gy.

Treatment was delivered on an Elekta HDR microSelectron unit. The whole process (from insertion to removal of applicators) was under general anesthesia. Patients were discharged on the same day after brachytherapy if all procedures went smoothly.

### 2.3. Follow Up

All patients had cervical biopsies at four quadrants at their fourth brachytherapy. If the biopsy was negative for malignancy, patients were reviewed at 8 weeks following the completion of whole treatment to assess response and toxicities. If the biopsy was positive, biopsies were repeated every 2 weeks to ensure complete remission after treatment. If malignant cells persisted, a pelvic MRI was performed for the consideration of salvage surgery. Patients were assessed every 3–4 months in the first 2 years, then every 6 months for the subsequent years.

### 2.4. Endpoint and Statistical Analysis

The primary endpoint was local control, defined as the absence of disease in the cervix and uterus, upper vagina and parametria on clinical examination, imaging and cervical biopsy.

The secondary endpoints were pelvic control, distant metastasis-free survival, overall survival (OS) and severe late toxicity rates. PC was defined as the absence of local and pelvic nodal disease. Distant metastasis-free survival was defined as any metastasis outside the pelvis, including the para-aortic region. OS was the date of diagnosis to date of death from any cause. Patients lost to follow-up were censored at the time of last follow-up. Severe, late toxicity was defined as G3-G5 complications (Common Toxicity Criteria v4.0) present at or after 91 days from the completion of treatment. The time to complete one brachytherapy session was counted.

The statistical analysis was conducted using SPSS software v28 (SPSS Inc., Chicago, IL, USA). Time to event analysis at 3 and 5 years was computed using the Kaplan–Meier method and log rank test. Univariable and multivariable analyses were conducted using the Cox regression model to assess the associations between the clinical outcomes and different variables, including stage, tumor size, nodal involvement, histology, use of chemotherapy, and HR-CTV volume and dosage. Variables with *p*-value < 0.1 in the univariable analysis were entered into multivariable analysis.

## 3. Results

A total of 135 patients with a median age of 56 years (range: 27–83) were included. The median follow-up time was 53.6 months (range: 3.0–99.6 months). The FIGO stage distribution was IB = 23 (17.0%), IIA = 20 (14.8%), IIB = 62 (45.9%), IIIA = 5 (3.7%), and IIIB = 25 (18.5%). A total of 78 patients (57.8%) had pelvic lymph node involvement and 3 (2.2%) had para-aortic lymph node involvement. A total of 107 patients (79.3%) had squamous cell carcinomas, 21 (15.6%) had adenocarcinomas and 7 (5.2%) had adenosquamous carcinomas. The median tumor width upon diagnosis assessed clinically was 4.8 cm (range 2–9.3 cm) and the median depth was 3.2 cm (range 0.8–4.6 cm). The clinical characteristics of the patients are summarized in Table 1.

### 3.1. Treatment

All patients completed the external beam RT as planned without any interruptions for treatment-related toxicity. A total of 132 patients (97.8%) were treated with pelvic RT alone and 3 patients (2.2%) had additional para-aortic RT. A total of 110 patients (81.5%) received concomitant chemotherapy, the most common regimen being weekly cisplatin 40 mg/m^2^ (104 patients, 77.0%). Six patients received concurrent, weekly carboplatin with AUC: 2. A total of 25 patients did not receive concomitant chemotherapy due to medical comorbidities or patient refusal.

All patients received CT-based IGABT, and all had pre-BT MRIs. In 35/135 patients (25.9%), a combined intracavitary/interstitial approach was used in at least one BT fraction. The mean HR-CTV was 42 *+/−* 8.9 cm^3^. The mean D90 (EQD2_10_) for HR-CTV was 84 Gy (range 67–104 Gy). For OAR, the mean D2cc for the bladder, rectum, sigmoid and bowel was 82 *+/−* 9.37 Gy, 66 *+/−* 6.61 Gy, 65 *+/−* 8.35 Gy and 61 *+/−* 8.94 Gy, respectively (all EQD2_3_). A summary of the brachytherapy treatment is presented in Table 2.

### 3.2. Clinical Outcomes

Table 3 shows the clinical outcomes of patients with cervical cancer treated with BT. The three- and five-year local control rates were both 90.7% (12/135 had local failures). A total of 7/135 patients (5.2%) had persistent disease after BT. For the seven patients with persistent disease, three patients also had pelvic or distant metastasis at the time of the last biopsy. Four patients managed to have salvage surgery and attained long survivorship (34.0–92.4 months). Five patients (3.7%) had true local recurrences, but none of them could successfully be rescued by salvage surgery. These five patients also had synchronous distant and pelvic nodal failures.

The three- and five-year pelvic control rates were both 84.3% (20/135 had pelvic failures). A total of 12 out of 20 pelvic failure patients (60%) had synchronous distant metastasis.

The three-year distant metastasis-free survival was 82.2% while the five-year distant metastasis-free survival was 80.0% (24/135 had distant metastasis). A total of 21 out of 24 patients (87.5%) received subsequent systemic treatment.

There were 14 deaths during the study period. The 3-year OS was 93.4% and 5-year OS was 87.2%. All patients died of cancer recurrence.

### 3.3. Prognostic Factors for Local Control, Pelvic Control, Distant Metastasis-Free Survival and Overall Survival Rates

Histology had a considerable impact on all four clinical outcomes: local control, pelvic control, distant metastasis-free survival and OS rates. Patients with adenocarcinomas had a lower local control rates (5-year local control rate: 74.8% vs. 95.1%, HR 5.82, 95% CI 1.84–18.34, *p* = 0.003); pelvic control rate (5-year pelvic control rate: 63.9% vs. 89.7%, HR 4.41, 95% CI 1.83–10.60, *p* = 0.001); distant metastasis-free survival (5-year distant metastasis-free survival: 67.2% vs. 86.5%, HR 2.83, 95% CI 1.17–6.84, *p* = 0.021) and OS (5-year OS: 69.3% vs. 92.7%, HR 4.38, 95% CI: 1.52–12.67, *p* = 0.003) than squamous cell carcinomas in both univariable and multivariable analyses. Histology was the only factor associated with local and pelvic controls (Supplementary Table S1a–d presents univariable and multivariable analyses).

More distant metastasis was observed in patients with HR-CTV volume > 30 cm^3^ (5-year distant metastasis-free survival: 76.6% vs. 85.5%, HR 3.44, 95% CI 1.18–9.42, *p* = 0.025) and presence of pelvic lymph node (5-year distant metastasis-free survival: 76.6% vs. 85.5%, HR 3.44, 95% CI 1.18–9.42, *p* = 0.025). OS was worse in patients without concurrent chemotherapy (5-year OS: 89.5% vs. 72.0%, HR 4.33, 95% CI: 1.40–13.33, *p* = 0.011).

### 3.4. Late Toxicities

Sixteen patients (11.9%) had documented long-term complications after BT. Nine patients (6.7%) had grade 3 or above complications, resulting in a 5-year risk of serious late morbidity of 5.9%. Two patients (1.5%) had grade 3 radiation cystitis with gross hematuria and needed blood transfusion. The EQD2 doses of D2cc bladder of these two patients were both less than 70 Gy. One patient (0.7%) had ureteric stricture and needed an operation. This patient was special as she had persistent disease after BT and had a salvage operation. Three patients (2.2%) had grade 3 radiation proctitis or enteritis. These three patients had a higher EQD2 dose of D2cc rectum and sigmoid, but still within the recommended dose constraints (Patient A: 73.1 Gy in rectum and 75.8 Gy in sigmoid; Patient B: 70.1 Gy in rectum and 73.4 Gy in sigmoid; Patient C: 70.1 Gy in rectum and 74.2 Gy in sigmoid). One patient (0.7%) had bowel perforation. One patient (0.7%) had vaginal stenosis and one (0.7%) had a rectovaginal fistula (Supplementary Table S2).

### 3.5. Time to Complete Brachytherapy Procedure

The mean duration of completion of one brachytherapy procedure was 142 min (range: 116–189 min). A total of 104 patients (77.0%) could be discharged on the same day of the procedure and did not need to stay in the in-patient ward overnight.

## 4. Discussion

The present study reported the long-term efficacy and toxicity data of CT-based BT for cervical cancer. The median follow-up of this study was 53.6 months, which was relatively long compared to other similar studies. The 5-year local control rate of 90.7% in our study compared well to 78–91% in other studies (Table 4: comparison to other studies). The treatment outcome was excellent, with 5-year pelvic control, distant-metastasis free survival and OS rates of 84.3%, 80.0% and 87.2%, respectively.

Several reports also reviewed the long-term results of CT-guided brachytherapy for cervical cancer. Tomizawa et al. retrospectively analyzed 221 patients with cervical squamous cell carcinomas treated with definitive radiotherapy using 3D-IGBT at the Gunma University Hospital (Maebashi, Gunma, Japan) [21]. The 5-year OS was 100.0% for T1b tumors, 82.5% (95% CI, 59.7–93.0%) for T2 tumors and 65.2% (95% CI, 45.4–79.2%) for T3 tumors. Zolciak-Siwinska et al. reviewed 216 patients with locally advanced cervical cancer treated with CT-guided brachytherapy [22]. The 5-year rates of OS and DFS were 66.4% and 58.5%, respectively. Ohno et al. reported the 5-year result of 93 patients with cervical cancer who underwent brachytherapy with CT guidance [23]. The 5-year local control, pelvic progression-free survival and OS rates were 94%, 90% and 86%, respectively. Most of the participants in these reports had squamous cell carcinomas of the cervix. Our study included a higher percentage of patients with adenocarcinomas (20.7%).

A large tumor size and HR-CTV D90 are well-known to be associated with poor local control [24]. In the RetroEMBRACE study, the factors correlated with local control were HR-CTV volume (HR: 1.017 per cm^3^, *p* = 0.004), HR-CTV D90 (HR: 0.967 per Gy, *p* = 0.022) and total treatment time (HR: 1.023 per day, *p* = 0.004) [9]. However, in our study, neither tumor size, HR-CTV D90 nor HR-CTV volume were associated with local control. The target volume contoured on CT is well-known to be larger than that contoured on MRI because of the poor definition of parametrial tumor infiltration on the CT [25]. CT, unlike MRI, does not clearly differentiate tumors, and it may overestimate the volume in patients with parametrial extensions upon diagnosis that have a good response to EBRT [26]. In our study, we followed the NRG Oncology consensus guidelines published in 2014 to contour the CTV, comprising the whole cervix and parametrial extension [18]. Compared to other similar MRI-based studies, the median HR-CTV volume was greater, which would result in a lower HR-CTV D90. This explained why the local control rate could still be achieved (5-year local control: 90.7%), even though the median dose of HR-CTV (84 Gy) was a bit lower than the recommended dose of 85/90 Gy [27,28,29].

Adenocarcinoma was significantly associated with inferior local control, pelvic control and OS. Growing evidence shows that adenocarcinomas behave differently to squamous cell carcinomas, with different patterns of metastatic recurrence and prognosis [30,31,32,33,34]. Previous studies demonstrated that adenocarcinomas have a worse prognosis and higher risk of distant metastasis compared to squamous cell carcinomas. Jung et al. observed that the mean OS for patients with squamous cell carcinomas was significantly longer than for patients with adenocarcinomas (276.6 months vs. 243.8 months, *p* = 0.0156) following a hysterectomy [35]. Eifel et al. reported that for tumors ≥ 4 cm in diameter, patients with adenocarcinomas had an estimated risk of death 1.9 times that of patients with squamous cell carcinomas (*p* < 0.01) [36]. Huang et al. observed that patients with adenocarcinomas appeared to have a shorter relapse-free survival than squamous cell carcinomas treated with RT [37]. A recently published study conducted by Liu et al. showed that, in patients with stage IIB-IV patients, the 5-year OS and disease-free survival rates were shorter in adenocarcinoma patients than squamous cell carcinoma (OS: 70.7% vs. 54.3%, *p* < 0.00; disease-free survival: 65.2% vs. 45.8%, *p* < 0.001) patients [38]. Our results are consistent with the above findings with an increased risk of distant metastasis and shorter OS in adenocarcinoma patients compared to squamous cell carcinoma patients.

While most of the previous studies focused on distant metastasis-free survival or OS rates, our study also confirmed that adenocarcinoma had worse local control. Adenocarcinoma is less sensitive to RT or chemotherapy. However, take note of the five patients with adenocarcinomas and persistent disease after brachytherapy; three patients managed to have salvage surgery and they all had long survival rates. This suggested a combined modality treatment may improve the outcome of patients with adenocarcinomas.

Although, at present, multiple international guidelines are still recommending the same treatment modalities for squamous cell carcinomas and adenocarcinomas, some suggested different treatment strategies for these two pathologies to improve the treatment outcomes [39,40]. Some studies proposed neoadjuvant chemotherapy using taxane and platinum-based chemotherapy before surgery for locally advanced cervical cancer [41,42]. Some preliminary data suggested better treatment responses with concurrent chemoradiotherapy using doublet chemotherapy with paclitaxel and cisplatin instead of single-agent cisplatin alone [43]. Studies on the addition of adjuvant chemotherapy to chemoradiotherapy have been conducted in the past two decades, but none have been practiced. A recently published meta-analysis, which included a total of 2150 patients, failed to show any survival or progression-free survival benefits of adding platinum-taxane chemotherapy following chemoradiotherapy [44]. The phase III RCT OUTBACK trial, which included 919 patients with locally advanced cervical cancer, adding four cycles of adjuvant carboplatin–paclitaxel after radical chemoradiotherapy did not improve the survival rates [45]. Another ACTLACC trial, including 259 patients with locally advanced cervical cancer, showed no significant improvement in response rates compared to chemoradiotherapy alone [46]. Ongoing studies are investigating if adding immune-checkpoint inhibitors will improve clinical outcomes (NCT02635360, NCT03192059). Moreover, there were some early reports on the use of carbon-ion RT for cervical cancer. For example, Wakatsuki et al. reported a manageable toxicity profile using carbon-ion RT for locally advanced adenocarcinoma of the cervix. Further studies are needed to confirm its therapeutic efficacy [47,48]. With the increasing incidence of cervix adenocarcinomas, which is also more aggressive and has a worse prognosis, future studies are warranted to investigate the best treatment strategies for this particular type of cancer.

There are several strengths presented in our study. First, we demonstrated excellent clinical outcomes with CT-based brachytherapy for cervical cancer. Second, the follow-up period in our study was relatively long, with a median follow-up of over four years. Third, we reported an efficient workflow for cervical cancer brachytherapy as most of the patients could complete their treatment in a day center without staying overnight. This workflow can set an example for other institutions without MRI-based brachytherapy planning and those with limited in-patient beds. However, several limitations existed: (1) we only included patients in a single institution; (2) 32 participants (23.7%) lost their follow-up since early January 2020 because of the COVID-19 pandemic (during the data analysis, these patients were censored and they did not affect the final outcome outcome); and (3) the late toxicity events were collected retrospectively from patients’ records. Some of the toxicity profiles, especially those with lower grades, might have been overlooked.

## 5. Conclusions

Although MRI-based brachytherapy is the gold standard of treatment for cervical cancer, our study demonstrated excellent long-term treatment outcomes with CT-guided brachytherapy. Our workflow using a day-center approach was feasible and can be considered by other institutions. Adenocarcinoma is a poor prognostic factor for all treatment outcomes. Future studies should focus on multi-modality strategies to improve the treatment outcomes of this particular type of cervical cancer.

## Figures and Tables

**Table 1:** Clinical characteristics of the participants.

Total	135 Patients	
	Median	Range
**Age**	56 years old	27–83 years old
**Follow-up period**	53.6 months	3.0–99.6 months
**Tumor characteristics**		
**Tumor width at diagnosis**	4.8 cm	2–9.3 cm
**Tumor depth at diagnosis**	3.2 cm	0.8–4.6 cm
**HPV-related**		
Yes	29	21.5%
No	7	5.2%
Unknown	99	73.3%
	**Number**	**Percentage**
**Stage**		
IB	23	17.0%
IIA	20	14.8%
IIB	62	45.9%
IIIA	5	3.7%
IIIB	25	18.5%
**Pelvic lymph node involvement**	78	57.8%
**Para-aortic lymph node involvement**	3	2.2%
**Type of carcinoma**		
Squamous cell carcinoma	107	79.3%
Adenocarcinoma/adenosquamous cell carcinoma	28	20.7%

**Table 2:** Summary of brachytherapy treatment.

		Number	Percentage
**Completed external beam radiotherapy**		135	100%
**Concurrent chemotherapy**		110	81.5%
**Brachytherapy**			
**Intracavitary only**		100	74.1%
**Intracavitary + interstitial needles**		35	25.9%
		**Mean ± SD**	**Range**
**Time to complete brachytherapy**		142 ± 17 min	116–189 min
**HR-CTV volume**		42 ± 8.9 cm^3^	20.5–65.6 cm^3^
**HR-CTV D90**		84 ± 7.54 Gy	67–104 Gy
**D2cc for**	**Bladder**	82 ± 9.37 Gy	55–105 Gy
	**Rectum**	66 ± 6.61 Gy	51–83 Gy
	**Sigmoid**	65 ± 8.35 Gy	47–80 Gy
	**Bowel**	61 ± 8.94 Gy	44–80 Gy

**Table 3:** Treatment outcomes of patients with brachytherapy for cervical cancer.

Event Outcome	Squamous Cell Carcinoma	%	Adeno-Carcinoma	%	Total	%	*p* *
	(*n* = 107)		(*n* = 28)		(*n* = 135)		
**Local failure**	5	4.70%	7	25.00%	12	8.90%	0.003
- Persistent disease	2	1.90%	5	17.90%	7	5.20%	
Salvage surgery	1	0.90%	3	10.70%	4	3.00%	
- Local recurrence after remission	3	2.80%	2	7.10%	5	3.70%	
**Pelvic failure**	10	9.30%	10	35.70%	20	14.80%	0.001
- Pelvic lymph node recurrence	5	4.70%	3	10.70%	8	5.90%	
**Distant failure**	15	14.00%	9	32.10%	24	17.80%	0.048
- Subsequent systemic treatment	14	13.10%	7	25.00%	21	15.60%	
**Death**	6	5.60%	8	28.60%	14	10.40%	0.002

* The *p*-values represent the comparison of the overall number of events for each survival outcome by histology.

**Table 4:** Comparison to other studies on IGABT for cervical cancer.

**Study**	**IGABT Technique**	**No. of Patients**	**Medan HR-CTV Volume (cm^3^)**	**Local Control**	**Overall Survival**
Present study	CT	135	42	90.7% (5-year)	87.2% (5-year)
Potter et al. [8]	MRI	1416	28	92% (5-year)	74% (5-year)
Potter et al. [11]	MRI	156	Mean tumor size > 5 cm	95% (3-year)	68% (3-year)
Charra-Brunaud et al. [12]	MRI	117	35.2	78.5% (2-year)	74% (2-year)
Sturdza et al. [9]	CT/MRI	731	37	89% (5-year)	65% (5-year)
Horne et al. [19]	MRI	239	31	90.8% (5-year)	72.7% (5-year)
Gill et al. [20]	CT/MRI	128	31	92% (2-year)	85% (2-year)
Horeweg et al. [10]	CT/MRI	155	Mean tumor size 4.6 cm	90.4% (5-year)	65.9% (5-year)

## Data Availability

The data presented in this study are available on request from the corresponding author. The data are not publicly available due to patients’ privacy.

## Supplementary Materials

The following supporting information can be downloaded at: https://www.mdpi.com/article/10.3390/cancers14163934/s1, Table S1a: Univariable and multivariable Cox regression analyses for local control; Table S1b: Univariable and multivariable Cox regression analyses for pelvic control; Table S1c: Univariable and multivariable Cox regression analyses for distant metastasis; Table S1d: Univariable and multivariable Cox regression analyses for overall survival; Table S2: Grades 3/4 late toxicities.
