# Supplementary material for: Treatment Outcomes of Computer Tomography-Guided Brachytherapy in Cervical Cancer in Hong Kong: A Retrospective Review
Source: Cancers (Basel). 2022 Aug 15;14(16):3934. doi: 10.3390/cancers14163934 (PMC9406104; doi:10.3390/cancers14163934)
Supplement: Supplementary file 1 [file cancers-14-03934-s001.zip › cancers-1868762-supplementary.pdf]

Supplementary Table S1a: Univariable and multivariable Cox regression analysis for local control

			Univariable				Multivariable			
	**no. of local failure**	**5-year local control**	HR	95% CI		p-value	HR	95% CI		p-value
Overall	12	90.7%	NA							
Factors										
Age			0.98	0.94	1.03	0.513				
Histology:										
- Squamous cell carcinoma	5	95.1%	Ref							
- Adenocarcinoma	7	74.8%	5.82	1.84	18.34	0.003	5.82	1.84	18.34	0.003
Tumor size			0.78	0.51	1.19	0.78				
Stage										
- I	4	81.3%	Ref							
- II	8	88.7%	0.68	0.20	2.24	0.522				
- III	0	100.0%	0	0	--	0.998				
Concurrent chemo										
- Yes	6	94.1%	Ref							
- No	6	89.9%	2.25	0.29	17.45	0.438				
HR-CTV vol										
- < 30 cm^3	4	91.2%	Ref							
- ≥ 30 cm^3	8	90.5%	1.09	0.33	3.60	0.893				
HR-CTV D90										
- ≥ 85 Gy	4	92.5%	Ref							
- < 85 Gy	8	89.4%	1.52	0.45	5.00	0.504				
Pelvic lymph node involvement										
- No	5	90.4%	Ref							
- Yes	7	90.1%	0.95	0.30	2.99	0.927				

Abbreviations: HR-CTV D90: dose delivered to 90% of high-risk clinical target volume; HR-CTV vol: volume of the high-risk clinical target volume ; NA: not available; Ref: reference

Supplementary Table S1b: Univariable and multivariable Cox regression analysis for pelvic control

	**No. of pelvic failure**	**5-year pelvic control**	**Univariable**				**Multivariable**			
Total	20	84.3%	HR	95% CI		p-value	HR	95% CI		p-value
Age			0.99	0.96	1.03	0.711				
Histology:										
- Squamous cell carcinoma	10	89.7%	Ref							
- Adenocarcinoma	10	63.9%	4.41	1.83	10.60	0.001	4.41	1.83	10.60	0.001
Tumor size			0.93	0.68	1.26	0.635				
Stage										
- I	7	68.6%	Ref			1.000				
- II	10	85.2%	0.47	0.18	1.24	0.126				
- III	3	91.8%	0.25	0.06	1.01	0.052				
Concurrent chemo										
- Yes	17	84.8%	Ref							
- No	3	84.0%	1.07	0.31	3.65	0.918				
HR-CTV vol										
- < 30 cm^3	6	86.4%	Ref							
- ≥ 30 cm^3	14	83.1%	1.25	0.48	3.26	0.645				
HR-CTV D90										
- ≥ 85 Gy	5	90.6%	Ref							
- < 85 Gy	15	79.7%	2.23	0.81	6.15	0.120				
Pelvic lymph node involvement										
- No	5	90.4%	Ref							
- Yes	15	79.2%	2.10	0.76	5.77	0.152				

Abbreviations: HR-CTV D90: dose delivered to 90% of high-risk clinical target volume; HR-CTV vol: volume of the high-risk clinical target volume; NA: not available; Ref: reference

Supplementary Table S1c: Univariable and multivariable Cox regression analysis for distant metastasis

	No. of distant metastasis	3-year distant metastasis-free survival	Univariable				Multivariable			
Total	24	80.0%	HR	95% CI		p-value	HR	95% CI		p-value
Age			1.02	0.99	1.06	0.18				
Histology:										
- Squamous cell carcinoma	15	86.5%	Ref							
- Adenocarcinoma	9	67.2%	2.33	1.02	5.33	0.045	2.83	1.17	6.84	0.021
Tumor size			1.12	0.86	1.46	0.392				
Stage										
- I	8	62.9%	Ref							
- II	8	88.5%	0.34	0.13	1.12	0.312				
- III	8	78.6%	0.63	0.24	1.68	0.354				
Concurrent chemo										
- Yes	18	68.5%	Ref							
- No	6	82.0%	2.07	0.82	5.24	0.124				
HR-CTV vol										
- < 30 cm^3	5	85.5%	Ref							
- $\geq$ 30 cm^3	19	76.6%	2.91	1.09	7.80	0.034	3.44	1.18	9.42	0.025
HR-CTV D90										
- $\geq$ 85 Gy	7	81.4%	Ref							
- < 85 Gy	17	77.1%	1.05	0.99	1.12	0.101				
Pelvic lymph node involvement										
- No	5	85.5%	Ref							
- Yes	19	76.6%	2.91	1.09	7.80	0.034	3.44	1.18	9.42	0.025

Abbreviations: HR-CTV D90: dose delivered to 90% of high-risk clinical target volume; HR-CTV vol: volume of the high-risk clinical target volume; NA: not available; Ref: reference

Supplementary Table S1d: Univariable and multivariable Cox regression analysis for overall survival

	**No. of death**	**3-year overall survival rate**	**Univariable**				**Multivariable**			
Total	14	87.2%	HR	95% CI		p-value	HR	95% CI		p-value
Age			1.03	0.99	1.08	0.169				
Histology:										
- Squamous cell carcinoma	6	92.7%								
- Adenocarcinoma	8	69.3%	4.98	1.73	14.38	0.003	4.38	1.52	12.67	0.003
Tumor size			0.88	0.59	1.30	0.507				
Stage										
- I	6	75.5%	1.00	1.00	1.00	1.000				
- II	4	93.9%	0.25	0.07	0.87	0.030				
- III	4	84.6%	0.44	0.12	1.549	0.200				
Concurrent chemo										
- Yes	9	89.5%	Ref							
- No	5	72.0%	3.68	1.24	11.02	0.020	4.33	1.40	13.33	0.011
HR-CTV vol										
- < 30 cm^3	3	85.9%	Ref							
- ≥ 30 cm^3	11	89.3%	1.66	0.46	5.99	0.436				
HR-CTV D90										
- ≥ 85 Gy	2	93.3%								
- < 85 Gy	12	83.9%	2.94	0.65	14.28	0.124				
Pelvic lymph node involvement										
- No	6	88.2%								
- Yes	8	88.6%	1.02	0.35	2.91	0.977				

Abbreviations: HR-CTV D90: dose delivered to 90% of high-risk clinical target volume; HR-CTV vol: volume of the high-risk clinical target volume; NA: not available; Ref: reference

Supplementary Table S2: Grade 3 / 4 late toxicities

	**Number**	**Percentage**
Total events	9	6.7%
Radiation cystitis	2	1.5%
Ureteric stricture	1	0.7%
Radiation proctitis/ enteritis	3	2.2%
Bowel perforation	1	0.7%
Vaginal stenosis	1	0.7%
Rectovaginal fistula	1	0.7%
